# Supplementary material for: Using multiple criteria for redesigning habitat corridor plans for Giant Pandas
Source: PLoS One. 2025 Jul 15;20(7):e0326792. doi: 10.1371/journal.pone.0326792 (PMC12262836; doi:10.1371/journal.pone.0326792)
Supplement: S1 File — S1 Table. TSS and AUC in each model in the ensemble model ± standard error. S2 Table. The length, elevation and other characteristics of potential giant panda habitat corridors. (DOCX) [file pone.0326792.s001.docx]

# Supporting Information

**Table S1. TSS and AUC in each model in the ensemble model ± standard error.**

| Accuracy test  Model algorithms | AUC | TSS |
| --- | --- | --- |
| Classification Tree Analysis | 0.959 ± 0.009 | 0.877 ± 0.012 |
| Flexible Discriminant Analysis | 0.976 ± 0.004 | 0.866 ± 0.012 |
| Generalized Boosting Model | 0.979 ± 0.003 | 0.894 ± 0.007 |
| Generalized Linear Models | 0.979 ± 0.004 | 0.894 ± 0.009 |
| Multivariate Adaptive Regression Splines | 0.981 ± 0.003 | 0.888 ± 0.011 |
| Random Forest | 0.983 ± 0.003 | 0.906 ± 0.009 |

**Table S2.** **The length, elevation and other characteristics of potential giant panda habitat corridors**

| ID | Path Length (m) | Elevation (m) | Forest Coverage (%) | Centrality | Distance to  Nature reserve (m) | Distance to  Residential area (m) | Weight set 1 | Weight set 2 | Weight set 3 |
| --- | --- | --- | --- | --- | --- | --- | --- | --- | --- |
| 1 | 5242 | 2234 | 1 | 525 | 0 | 3765 | 0.836 | 0.804 | 0.371 |
| 2 | 3828 | 1724 | 1 | 329 | 0 | 2072 | 0.879 | 0.832 | 0.365 |
| 3 | 8828 | 1560 | 1 | 793 | 0 | 3088 | 0.883 | 0.831 | 0.412 |
| 4 | 3414 | 1638 | 1 | 659 | 0 | 2169 | 0.914 | 0.867 | 0.415 |
| 5 | 22414 | 2023 | 1 | 187 | 1402 | 2748 | 0.610 | 0.551 | 0.215 |
| 6 | 10000 | 2000 | 1 | 291 | 0 | 3026 | 0.783 | 0.739 | 0.316 |
| 7 | 12000 | 3630 | 1 | 488 | 48790 | 12146 | 0.441 | 0.426 | 0.185 |
| 8 | 23828 | 3419 | 0.73 | 683 | 41946 | 4607 | 0.000 | 0.000 | 0.000 |
| 9 | 15000 | 1677 | 1 | 328 | 5352 | 1910 | 0.714 | 0.652 | 0.278 |
| 10 | 9485 | 1334 | 1 | 988 | 193 | 2202 | 0.897 | 0.837 | 0.435 |
| 11 | 11313 | 1981 | 1 | 291 | 0 | 3006 | 0.770 | 0.723 | 0.308 |
| 12 | 19828 | 2909 | 1 | 564 | 678 | 2753 | 0.544 | 0.511 | 0.234 |
| 13 | 17970 | 3792 | 0.54 | 925 | 792 | 7213 | 0.168 | 0.240 | 0.140 |
| 14 | 5000 | 2576 | 1 | 836 | 2504 | 1341 | 0.744 | 0.717 | 0.361 |
| 15 | 3000 | 3203 | 1 | 1412 | 562 | 3638 | 0.768 | 0.767 | 0.444 |
| 16 | 19000 | 3152 | 1 | 1333 | 5644 | 2971 | 0.533 | 0.509 | 0.312 |
| 17 | 13414 | 3414 | 0.97 | 639 | 2577 | 4897 | 0.563 | 0.558 | 0.264 |
| 18 | 2414 | 3056 | 1 | 1557 | 0 | 8773 | 0.908 | 0.913 | 0.530 |
| 19 | 5242 | 3330 | 1 | 1207 | 199 | 6619 | 0.775 | 0.779 | 0.429 |
| 20 | 6000 | 2692 | 1 | 639 | 0 | 15343 | 1.000 | 1.000 | 0.477 |
| 21 | 3828 | 2245 | 1 | 342 | 5712 | 3042 | 0.794 | 0.760 | 0.331 |
| 22 | 1414 | 1953 | 1 | 332 | 8885 | 2053 | 0.825 | 0.782 | 0.341 |
| 23 | 3414 | 1996 | 1 | 639 | 8885 | 2006 | 0.808 | 0.766 | 0.365 |
| 24 | 3828 | 2483 | 1 | 983 | 4973 | 3065 | 0.798 | 0.772 | 0.403 |
| 25 | 5242 | 2125 | 1 | 1575 | 8967 | 1623 | 0.806 | 0.768 | 0.461 |
| 26 | 2828 | 1912 | 1 | 332 | 5951 | 912 | 0.808 | 0.762 | 0.331 |
| 27 | 2414 | 2517 | 1 | 2889 | 9629 | 4480 | 0.904 | 0.889 | 0.654 |
| 28 | 6242 | 2457 | 1 | 6083 | 13146 | 1579 | 0.943 | 0.929 | 1.000 |
| 29 | 19798 | 2220 | 1 | 935 | 5924 | 2030 | 0.611 | 0.559 | 0.295 |
| 30 | 4828 | 1879 | 1 | 3052 | 7336 | 1676 | 0.927 | 0.891 | 0.672 |
| 31 | 21213 | 1976 | 1 | 963 | 7965 | 1890 | 0.613 | 0.553 | 0.295 |
| 32 | 5242 | 3333 | 0.91 | 3296 | 0 | 4587 | 0.762 | 0.785 | 0.646 |
| 33 | 4828 | 2936 | 1 | 2559 | 2799 | 5174 | 0.856 | 0.852 | 0.602 |
| 34 | 7000 | 3028 | 1 | 3201 | 0 | 7823 | 0.917 | 0.922 | 0.702 |
| 35 | 22556 | 1988 | 1 | 1387 | 1160 | 2882 | 0.676 | 0.621 | 0.371 |
| 36 | 23213 | 2458 | 1 | 389 | 17894 | 4219 | 0.483 | 0.429 | 0.177 |
| 37 | 9242 | 1664 | 1 | 1586 | 8479 | 2588 | 0.843 | 0.791 | 0.474 |
| 38 | 20384 | 2108 | 1 | 1604 | 5420 | 2179 | 0.657 | 0.605 | 0.386 |
| 39 | 4000 | 2733 | 1 | 1395 | 725 | 2065 | 0.787 | 0.770 | 0.444 |
| 40 | 14000 | 2240 | 1 | 4076 | 3105 | 2841 | 0.864 | 0.836 | 0.750 |
| 41 | 9000 | 1311 | 1 | 1232 | 4588 | 1711 | 0.882 | 0.820 | 0.452 |
| 42 | 16556 | 1560 | 1 | 3101 | 8865 | 1684 | 0.821 | 0.764 | 0.615 |
| 43 | 10828 | 1908 | 1 | 1333 | 4877 | 1572 | 0.779 | 0.730 | 0.418 |
| 44 | 5414 | 2847 | 1 | 647 | 5 | 7457 | 0.829 | 0.820 | 0.392 |
| 45 | 15485 | 3112 | 0.96 | 1289 | 2527 | 8036 | 0.665 | 0.660 | 0.380 |
| 46 | 6414 | 2176 | 1 | 1079 | 10140 | 3643 | 0.794 | 0.757 | 0.405 |
| 47 | 3414 | 2875 | 1 | 679 | 6396 | 4861 | 0.762 | 0.748 | 0.360 |
| 48 | 4828 | 3293 | 1 | 1147 | 11560 | 3574 | 0.655 | 0.646 | 0.359 |
| 49 | 9071 | 2866 | 1 | 979 | 20130 | 3639 | 0.604 | 0.575 | 0.308 |
| 50 | 5828 | 2068 | 1 | 1266 | 13153 | 2140 | 0.777 | 0.735 | 0.414 |
| 51 | 2828 | 2379 | 1 | 792 | 0 | 2974 | 0.842 | 0.817 | 0.405 |
| 52 | 18556 | 1873 | 1 | 1341 | 7666 | 2440 | 0.691 | 0.633 | 0.372 |
| 53 | 17000 | 2616 | 1 | 1469 | 23051 | 3856 | 0.552 | 0.510 | 0.326 |
| 54 | 4000 | 1763 | 1 | 894 | 1377 | 1746 | 0.885 | 0.840 | 0.426 |
| 55 | 21142 | 1671 | 1 | 961 | 6355 | 2789 | 0.683 | 0.617 | 0.326 |
| 56 | 23656 | 2158 | 1 | 424 | 11523 | 2792 | 0.529 | 0.468 | 0.199 |
| 57 | 12000 | 2195 | 1 | 2235 | 23042 | 5057 | 0.732 | 0.690 | 0.491 |
| 58 | 23071 | 1699 | 1 | 1344 | 32964 | 2151 | 0.505 | 0.424 | 0.272 |
| 59 | 6000 | 1874 | 1 | 1073 | 29222 | 2547 | 0.706 | 0.649 | 0.353 |
| 60 | 13727 | 1832 | 1 | 1094 | 37488 | 3245 | 0.584 | 0.515 | 0.290 |
| 61 | 8828 | 1932 | 1 | 533 | 34140 | 2745 | 0.612 | 0.550 | 0.250 |
| 62 | 10414 | 2181 | 1 | 999 | 26162 | 4853 | 0.670 | 0.622 | 0.332 |
| 63 | 15727 | 2063 | 1 | 565 | 32162 | 2861 | 0.526 | 0.460 | 0.210 |
| 64 | 16656 | 1871 | 1 | 1480 | 42627 | 1843 | 0.503 | 0.429 | 0.288 |
| 65 | 23727 | 3552 | 1 | 509 | 11849 | 5110 | 0.387 | 0.365 | 0.158 |
| 66 | 17970 | 2552 | 1 | 665 | 23566 | 2600 | 0.482 | 0.431 | 0.206 |
| 67 | 25000 | 3000 | 1 | 709 | 28374 | 2218 | 0.301 | 0.251 | 0.124 |
| 68 | 10071 | 4122 | 0.76 | 243 | 15792 | 9673 | 0.330 | 0.379 | 0.138 |
| 69 | 19384 | 2519 | 0.59 | 611 | 35268 | 1575 | 0.036 | 0.037 | 0.011 |
| 70 | 22000 | 2297 | 0.93 | 946 | 10515 | 1937 | 0.487 | 0.443 | 0.240 |
| 71 | 22627 | 3058 | 1 | 611 | 0 | 3432 | 0.509 | 0.479 | 0.224 |
| 72 | 24242 | 2244 | 0.82 | 354 | 4134 | 1715 | 0.379 | 0.349 | 0.134 |
